# Antemortem diagnosis of human rabies: A case report

**DOI:** 10.1002/ccr3.3615

**Published:** 2020-12-05

**Authors:** Wael Goravey, Ahmed Husain, Gawahir A. Ali, Muna A. Al Maslamani, Hisham Ziglam

**Affiliations:** ^1^ Department of Infectious Diseases Communicable Diseases Centre Hamad Medical Corporation Doha Qatar

**Keywords:** antemortem, encephalitis, rabies, real‐time polymerase chain reaction, saliva

## Abstract

Antemortem diagnosis of rabies is challenging, and usually, more than one test modality is needed to confirm the diagnosis. No effective treatment exists so far, and the Milwaukee Protocol is debatable.

## Graphical abstract

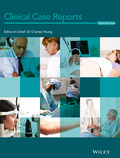

## 1 INTRODUCTION

Rabies is zoonotic disease and can cause progressive encephalitis and death. Antemortem diagnosis is challenging, and treatment is supportive. We report a young patient presented with classic furious rabies; the diagnosis was confirmed by real‐time polymerase chain reaction assays on saliva samples.

Rabies is one of the most fearful neglected human diseases belonging to the family Rhabdoviridae, genus Lyssavirus, and it is a single‐strand RNA virus.^1^ The estimated worldwide death of this vaccine‐preventable zoonotic disease is 24 000‐93 000 victims per year, and most of them are young in Africa and Asia.^2^

Most rabies infection is transmitted by exposure to saliva from an animal bite, mainly carnivores or bats with a variable incubation period. The presentation of rabies once the rabies viruses (RABV) established in the nervous system can be either encephalitic or paralytic rabies with a tendency toward rapid progression and death.^3^

Antemortem diagnosis of rabies requires several specimens and many laboratory modalities due to the limited sensitivity of a single test. Postmortem detection of fluorescent antibody test (FAT) on a brain sample remains the gold standard test for rabies diagnosis, despite challenges. Real‐time PCR can be used for antemortem and postmortem diagnosis of rabies with high sensitivity even in samples with low viral load.^4^ There is no known effective treatment for rabies, and the disease is invariably fatal.^5^

We report the present patient, who was diagnosed as RABV infection and managed accordingly.

## 2 CASE DESCRIPTION

A 33‐year‐old male with no chronic illness is presented to the emergency department, Hamad General Hospital, Doha, Qatar, with 2 days of history of fever, sore throat, irritability, and inability to drink water. He came one month ago from his home country to join a new job. The patient recalled a history of biting to his right ear by an unknown animal 3 months ago. No medical advice was sought and reported a completely healed wound. On examination, appeared agitated, febrile with a temperature of 38.3°C, and stable other vitals. Anxiety was noted when water was mentioned or seen by the patient. There is no nuchal rigidity or any focal neurological signs. A healed scar was noted in the pinna of the right ear. Blood tests, including complete blood counts, renal profile, liver function test, and C‐reactive protein, were within the reference ranges. No abnormalities were detected in the CT brain. The lumbar puncture showed lymphocytic CSF with negative bacterial cultures. Intravenous ceftriaxone and acyclovir were started to cover for possibilities of viral or bacterial encephalitis, but both stopped latter when the CSF bacterial cultures and viral PCR panel came negative. Rabies real‐time PCR for CSF and nape of skin biopsy were all negative. There was no rabies virus (RABV) neutralizing antibodies detectable in the CSF and plasma; however, two different real‐time PCR assays for RABV on saliva samples tested positive.^6^, ^7^ Immediately on suspicion of rabies, the patient received rabies vaccine, rabies immune globulin, and ribavirin. On day 3, the patient was intubated and sedated after he had developed hypotension, desaturation, and decreased level of consciousness. He continued to deteriorate at day 7 with generalized convulsions and further hypotension despite pressure support until sustained uncontrolled hypotension on day 10. Brainstem death was confirmed on day 18 where the family accepted to stop all forms of treatment including ventilatory support and shortly after succumbed to the illness.

## 3 DISCUSSION

Rabies is a zoonotic disease and potentially vaccine‐preventable. Despite the existence of safe and effective anti‐rabies vaccines, cost and awareness were the major constraints in Asia and Africa.^8^

In the Arabian Peninsula, RABV is enzootic with human cases of RABV, which has been reported previously in Qatar.^9^ The vaccination status of the host, proximity of the bite to the brain, the inoculum size, and the virulence of RABV strain are the major determinants of the incubation period which varies from days to years.^5^ Most rabies infection is transmitted by exposure to saliva from an animal bite, mainly carnivores or bats. Clinically, RABV infection is present in 2 distinct major forms, and around 65% will develop the furious type and the remaining paralytic rabies. Other forms of presentation include multi‐organ involvement, renal failure, acute respiratory distress syndrome, pericarditis, and myocarditis with complete heart block.^5^ Also, agitation, confusion, signs of autonomic dysfunction, and hydrophobia (pathognomonic) and aerophobia were all reported. Phobic spasms in response to tactile, auditory, visual, or olfactory stimuli pose high moralities within days without intensive ventilatory support. The pathophysiology for the characteristic aggressive behavior in the encephalitic form of rabies remains unknown.^10^ Our patient is a young male who recently arrived in Qatar with unidentified animal bite 3 months before arrival with no history of postexposure vaccination or rabies immune globulin administration. He presented with typical symptoms of rabies encephalitis and hydrophobia. These make the diagnosis of rabies encephalitis high in the list of our differential diagnoses, but we were opting to cover for viral and bacterial encephalitis and consider other possibilities like toxic or metabolic encephalopathy pending the work‐up.

There are no routine sensitive tests for antemortem diagnosis, despite advances made in understanding the virus behaviors. Detection of virus antigen in brain tissue remains the gold standard diagnostic technique with almost 100% sensitivity. Hemi‐nested polymerase chain reaction (RT‐hnPCR) showed high sensitivity and specificity irrespective of the time to clinical symptoms or sample collection. The downside of RT‐hnPCR is not routinely available.^4^ The negative serum and CSF neutralizing antibodies against the virus up to the tenth day after symptoms limit the use of the test for assessing antibody response after vaccination rather than the diagnostic role which explains the negativity of the tests in our patient as the sample was taken two days into the symptoms.^11^ In our case, the negativity of the PCR tests in CSF and nape of skin biopsy did not preclude the diagnosis of rabies since the clinical suspicion was high and can be elucidated by the absence of viremia, intermittent virus shedding, time of the sample collection, and transportation or storage of the samples.^4^ There are no specific findings for rabies in imaging studies compared with other viral encephalitides. However, MRI findings of frontotemporal hyperintense signals may be seen.^12^ There is no documented medical treatment for clinical rabies so far. The aim is to prevent the development of rabies by wound care and postexposure prophylaxis with hyperimmune rabies serum and active immunization. These interventions reduce the mortality risk from 37% to 60% to almost zero following a bite from a rabid animal.^13^ Many factors influence the adoption of an aggressive management protocol for confirmed or suspected rabies infection. Besides, awaiting or unavailability of the rabies tests in the early stage of the illness should initiate the aggressive treatment without delay.^10^ Inducing coma and treating with antiviral drugs to allow the native immune response to mature is the basis of the debated Milwaukee Protocol.^14^ Ribavirin and amantadine were used as antiviral strategies, while coma was induced by ketamine and midazolam in the original Milwaukee Protocol.^15^ Looking at the young age of the patient and the benefits of the doubts, we decided to adopt initially the aggressive approach. The patient received a rabies vaccine, and in the context of encephalitic symptoms, ceftriaxone and acyclovir were added to cover for possibilities of bacterial and viral encephalitis, respectively. Furthermore, ribavirin added, and ketamine was used to induce the coma as recommended in the Milwaukee Protocol. Despite these measures, the patient continues to deteriorate and eventually died. Infection control precautions were practiced by all healthcare workers caring for the patient.

## 4 CONCLUSION

RABV infection is a fatal disease, and prompt diagnosis requires a high index of suspicion as initial laboratory tests might be negative for rabies. Despite advances in medicine, RABV infection is still a worldwide health threat to humankind with the greatest mortality rate among all viral encephalitis. Antemortem diagnosis is very challenging with no up‐to‐date effective therapy.

## CONFLICT OF INTEREST

Authors declared no competing interests about the current publication.

## AUTHOR CONTRIBUTIONS

WG: performed corresponding author, data acquisition, and manuscript preparation. AH and GA: contributed to data acquisition and manuscript writing. MA and HZ: supervised all the aspects and contributed to final manuscript editing.

## Funding information

Open access funding was provided by the Qatar National Library.

## Data Availability

The authors confirm that the datasets supporting the findings of this case are available from the corresponding author upon request.
